# The Treatment of Syphilis by Hypodermic Injections

**Published:** 1887-11

**Authors:** 


### *THE TREATMENT OF SYPHILIS BY HYPODERMIC INJECTIONS.*

Dr. Prince A. Morrow, in the *Medical Record*, gives the results of his observations on the treatment of syphilis by hypodermic injections of mercury, as practical in the male and female syphilitic hospitals of Paris—the Louraine and the DuMidi.

After describing the methods of treatment by both the soluble and insoluble mercurials, and the relative merits and disadvantages of each, and the claims made for each by their respective champions, he concludes with the following summary:

"The hypodermic use of mercury, in simplicity, convenience, scientific accuracy, rapidity of action and the development of a maximum effect from a minimum quantity, constitutes a decided improvement over the ordinary modes of introducing this drug into the system.

"The hypodermic method is not as liable to cause salivation, gastro-intestinal disorders and other toxic symptoms of hydrargyrism.

"There is a remarkable unanimity of opinion as to its efficacy in suppressing the active manifestations of the secondary stage, and hastening their involution.

"The claim that the hypodermic use of mercury increases its potentiality and widens the range of its curative action, enabling it not only to subdue refractory secondary lesions, which visit ordinary treatment, but also tertiary lesions of syphilis, may be considered as yet *sub judice*. The claim that it endows mercury with a greater permanence of action, preventing relapses and preserving the patient from manifestations of the diathesis for a long period, is not proven.

"The more portentious claims that the hypodermic injection of twenty-five centigrammes of the bichloride, or forty centigrammes of calomel, cures syphilis, must be rejected as extravagant and absurd.

"The irritant action of mercury introduced subcutaneously, manifest in the production of pain and local accidents, renders its general employment in the systematic treatment of syphilis impracticable.

"Until these objectionable features be modified or overcome, the proper position of hypodermic medication in the therapeutics of syphilis is in the category of adjuvants.

"Its employment is indicated where the necessities of the case demand a rapid and intense mercurialization. In certain emergencies, where, for example, the integrity of an important organ is threatened, its prompt and energetic action renders it superior to other modes of mercurialization.

"In exceptional cases, marital syphilis, for example, where the exigencies of the situation demand secrecy in treatment with the speediest possible suppression of the symptoms, this method is to be recommended.

"In cases where gastro-intestinal irritation is so marked as to forbid the introduction of mercury to the stomach, it constitutes a most efficient reserve treatment.

"In tertiary syphilis, where the iodine idiosyncrasy is so marked as to preclude the use of iodide of potassium, the hypodermic use of mercury should be substituted.

"My own impression is, that while the hypodermic will never supplant the classic modes of employing mercury, yet it constitutes a decided acquisition to our therapeutical resources against syphilis, too valuable to be ignored or practically disregarded, as has been the case in this country."

